# Introducing a proline in the α1 M2-M3 linker relieves a molecular brake on channel activation in α1β2γ2 GABA_A_ receptors

**DOI:** 10.1371/journal.pone.0357367

**Published:** 2026-09-08

**Authors:** Netrang G. Desai, Pranavi Garlapati, Cecilia M. Borghese, Marcel P. Goldschen-Ohm

**Affiliations:** Department of Neuroscience, University of Texas at Austin, Austin, Texas, United States of America; University of Illinois at Urbana-Champaign, UNITED STATES OF AMERICA

## Abstract

GABA_A_ receptors (GABA_A_Rs) are pentameric ligand-gated ion channels (pLGICs) essential for inhibitory synaptic transmission throughout the central nervous system. Despite progress in understanding their three-dimensional structure, the molecular basis for how neurotransmitter binding is transduced to ion channel gating remains poorly understood. Furthermore, relatively little is known about the contributions of distinct subunits to this coupling within typical heteromeric receptors. A highly conserved proline (site 1) in the M2-M3 linker of pLGIC subunits is involved in channel gating – e.g., P273 in the GABA_A_R β2 subunit. In GABA_A_Rs, only the β subunits have an additional proline in the M2-M3 linker (site 2) – e.g., β2(P276) – whereas all other subunits have a non-proline at the homologous site 2 position. Here, we investigate the functional contribution of proline at site 2 in distinct subunits of α1β2γ2 GABA_A_Rs. We expressed wild type or mutant α1β2γ2 GABA_A_Rs in *Xenopus laevis* oocytes and used two-electrode voltage clamp electrophysiology to record channel currents in response to GABA and/or other ligands. First, we introduced a proline at site 2 in α1 or γ2 subunits: α1(A280P) and γ2(S291P). Second, we replaced the site 2 proline in the β2 subunit with its homologous non-proline residue from α1 or γ2 subunits: β2(P276A) or β2(P276S). We show that α1(A280P) confers enhanced GABA-sensitivity and spontaneous unliganded channel activity, whereas γ2(S291P) has minor effects on channel activation. In contrast, β2(P276A) or β2(P276S) either had no effect or enhanced GABA-activation, respectively, indicating complex functional dependence on the side chain at site 2 in the β2 subunit. When in combination with other substitutions, the presence or absence of α1(A280P) was consistently correlated with enhanced GABA-sensitivity and spontaneous unliganded opening. Thus, introduction of a proline at site 2 in the α1 M2-M3 linker biases the channel towards an activated state and prevents it from remaining closed at rest.

## Introduction

**Fig 1:**
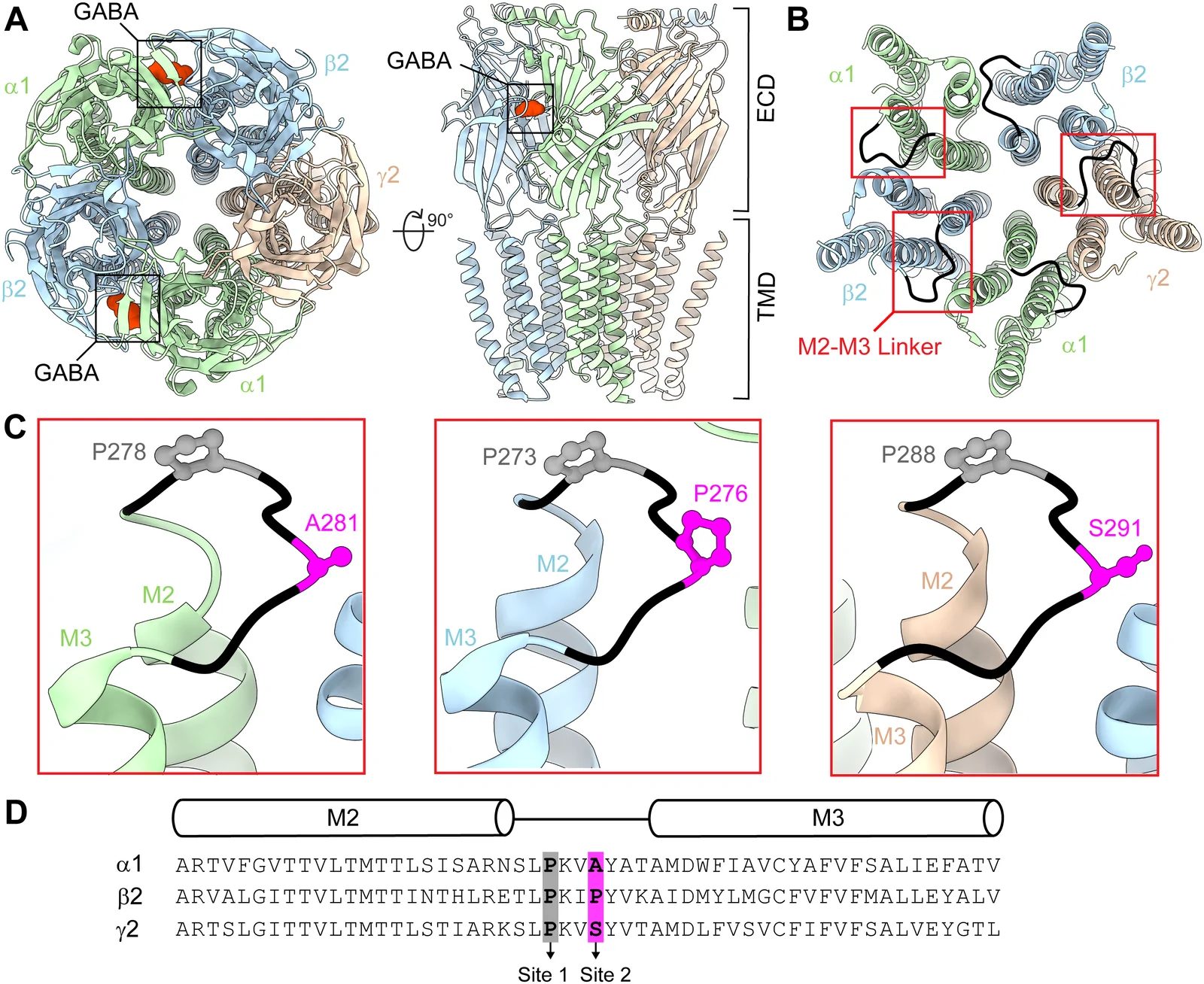
M2-M3 linker prolines in a synaptic α1β2γ2 GABA_A_R. (A) Cryo-EM map (PDB: 6X3X) of a human synaptic α1β2γ2 GABA_A_R in complex with GABA (red) viewed from the top (left) and side (right). (B) Same view as in panel A for the transmembrane domain only. The M2-M3 linkers are colored black. (C) Zoom in of boxed regions in panel B. A highly conserved proline at site 1 in α1, β2, and γ2 subunits is highlighted in grey. A proline at site 2 found in β subunits only and the homologous non-proline residues in α1 and γ2 subunits are highlighted in magenta. The mature protein numeration for β2 and γ2 subunits is the same for rat and human, but the numeration for the rat α1 subunit is (human numeration −1) for most of the subunit. (D) Sequence alignment of the M2-M3 linker regions (black line) for rat α1, β2, and γ2 subunits. Transmembrane helices M2 and M3 are indicated with cylinders above the alignment. Structures visualized using ChimeraX [14].

For GABA_A_ receptors (GABA_A_Rs) and related pentameric ligand-gated ion channels (pLGICs), coupling of neurotransmitter binding in the extracellular domain (ECD) to gating of the ion channel pore in the transmembrane domain (TMD) involves several loops located at the ECD-TMD interface (Fig 1A) [1–5]. One of these loops, the M2-M3 linker, connects the second and third transmembrane domains, and interacts with ECD loops (Fig 1B) [1, 2, 6–13]. Studies have uncovered asymmetric functional effects of M2-M3 linker perturbations in distinct subunits within typical heteromeric synaptic α1β2γ2 GABA_A_Rs [6, 7, 10, 13]. However, the physical basis underlying this asymmetry remains to be fully understood.

**Fig 2:**
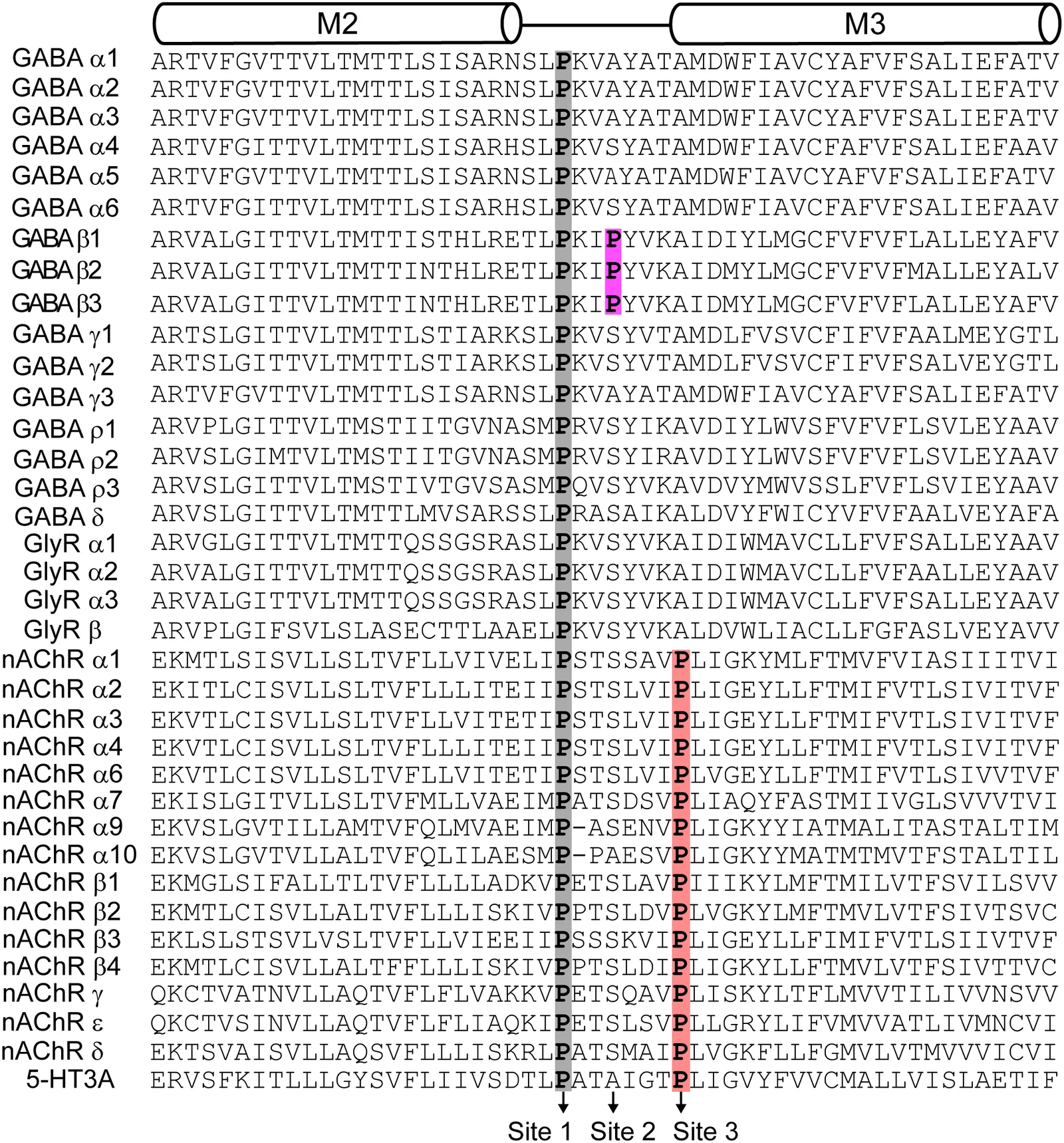
Sequence alignment of M2-M3 transmembrane region for pLGICs. Transmembrane helices M2 and M3 are indicated with cylinders above the alignment. A conserved proline at site 1 across the subunits of pLGIC receptors is highlighted in grey. A proline at site 2 conserved only in β subunits of GABA_A_Rs is highlighted in magenta. A proline found at site 3 in nAChR and 5-HT_3A_ subunits is highlighted in salmon. All sequences are from rat, which are very similar to those from humans. Importantly, the overall site 1-3 proline pattern is the same in human subunits.

All pLGICs have a highly conserved proline in the M2-M3 linker at site 1 which is involved in channel gating (Figs 1C,D and 2). Early studies of nicotinic acetylcholine receptors (nAChRs) suggested that a “pin-in-socket” mechanism involving this conserved proline contributes to coupling agonist binding to pore opening [15–22]. Consistent with this idea, substitutions of this proline in homomeric α7 nAChR result in non-functional channels [23]. However, substitutions in other pLGICs such as GABA_A_Rs and 5-HT_3A_ receptors do not abolish channel function, indicating that such a mechanism is not requisite in these channels [23–25]. Nonetheless, substitutions of this conserved proline in either α1 or β2 subunits of GABA_A_Rs do alter channel gating properties [24, 25], indicating that this proline at least contributes to channel activity.

In addition to the highly conserved site 1 proline, cationic nAChRs and 5-HT_3A_ receptors have another proline located at the top of the M3 transmembrane helix (site 3) (Fig 2). One of the proposed mechanisms for gating is the cis-trans isomerization of this site 3 proline which strongly correlates with the channel activation of 5-HT_3A_ receptors [26]. Although GABA_A_Rs lack a homologous proline at site 3, they do have an additional proline in the M2-M3 linker which is specific to β subunits only (site 2) (Figs 1C,D and 2). Here, we investigate the functional contribution of proline at site 2 in each distinct subunit within typical synaptic α1β2γ2 GABA_A_Rs. We show that introduction of a proline at site 2 in the α1 subunit enhances GABA-sensitivity and prevents the channel from remaining closed at rest. The role of proline at site 2 in the β2 subunit is more ambiguous, with differential effects for alanine or serine substitutions. Nonetheless, across tested combinations of substitutions in multiple subunits, enhanced GABA-sensitivity and spontaneous activity consistently correlated with the presence of a proline at site 2 in the α1 subunit. This suggests that the presence or absence of a proline-induced kink in the α1 M2-M3 linker at site 2 is an important determinant of channel activation. Our observations provide new insight into the subunit-specific effects of M2-M3 linker perturbations on the channel gating of α1β2γ2 GABA_A_Rs.

## Results

### Introducing a proline at site 2 in the α1 M2-M3 linker enhances channel activation

**Fig 3:**
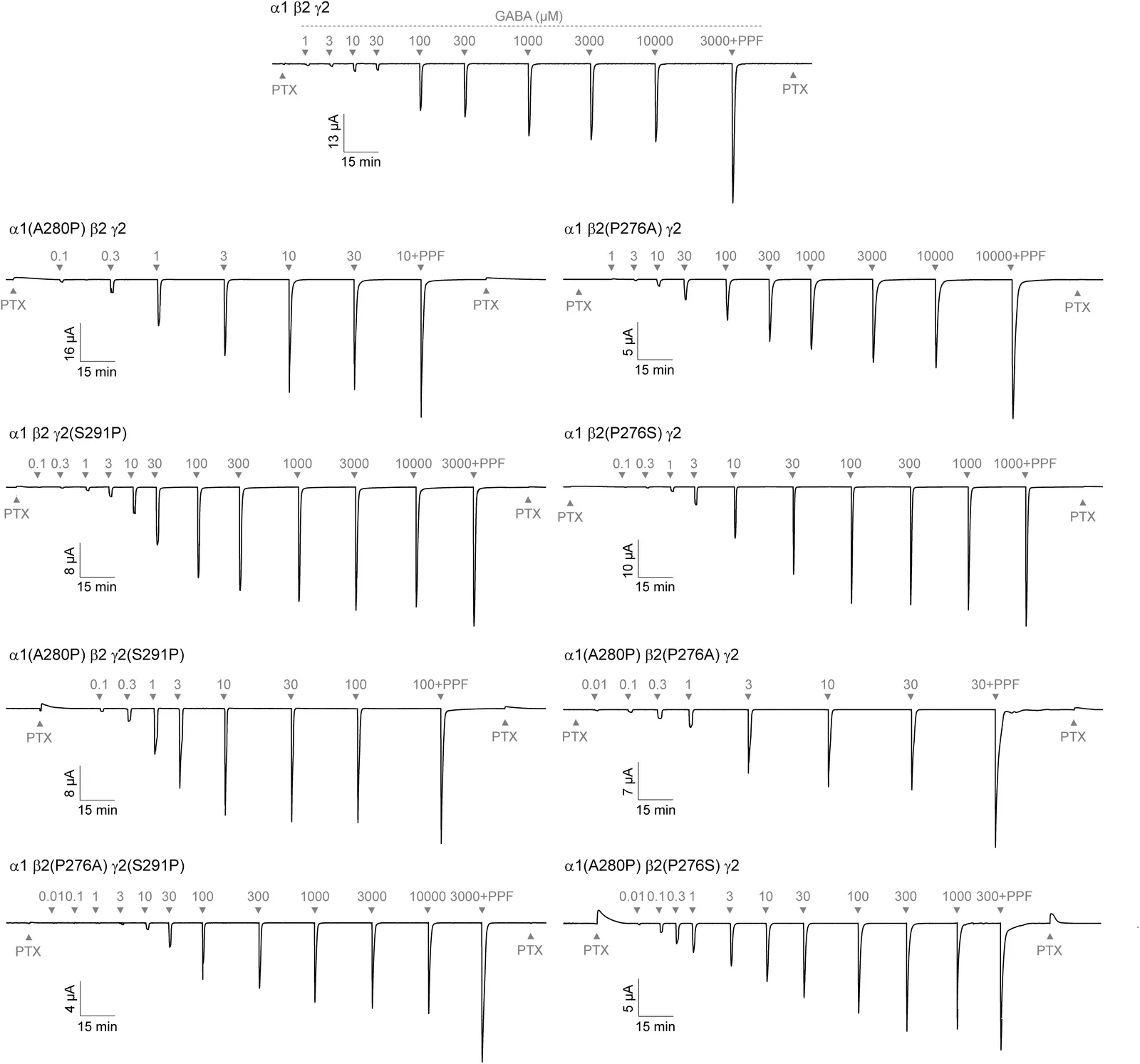
Representative current tracings of wild-type and mutant α1β2γ2 GABA_A_Rs. Example two-electrode voltage clamp recordings of wild-type and mutant α1β2γ2 GABA_A_Rs. A 10 s pulse of 1 mM picrotoxin (PTX; to assess for unliganded opening) was followed by a series of 20-40 s pulses (sufficient to resolve peak) of increasing concentrations of GABA (µM), a 20 s pulse of saturating GABA in combination with 30 µM propofol (PPF) to estimate the maximal possible current, and a final 10 s pulse of 1 mM PTX. Labeled numeric values for pulses indicate GABA concentration (µM).

**Fig 4:**
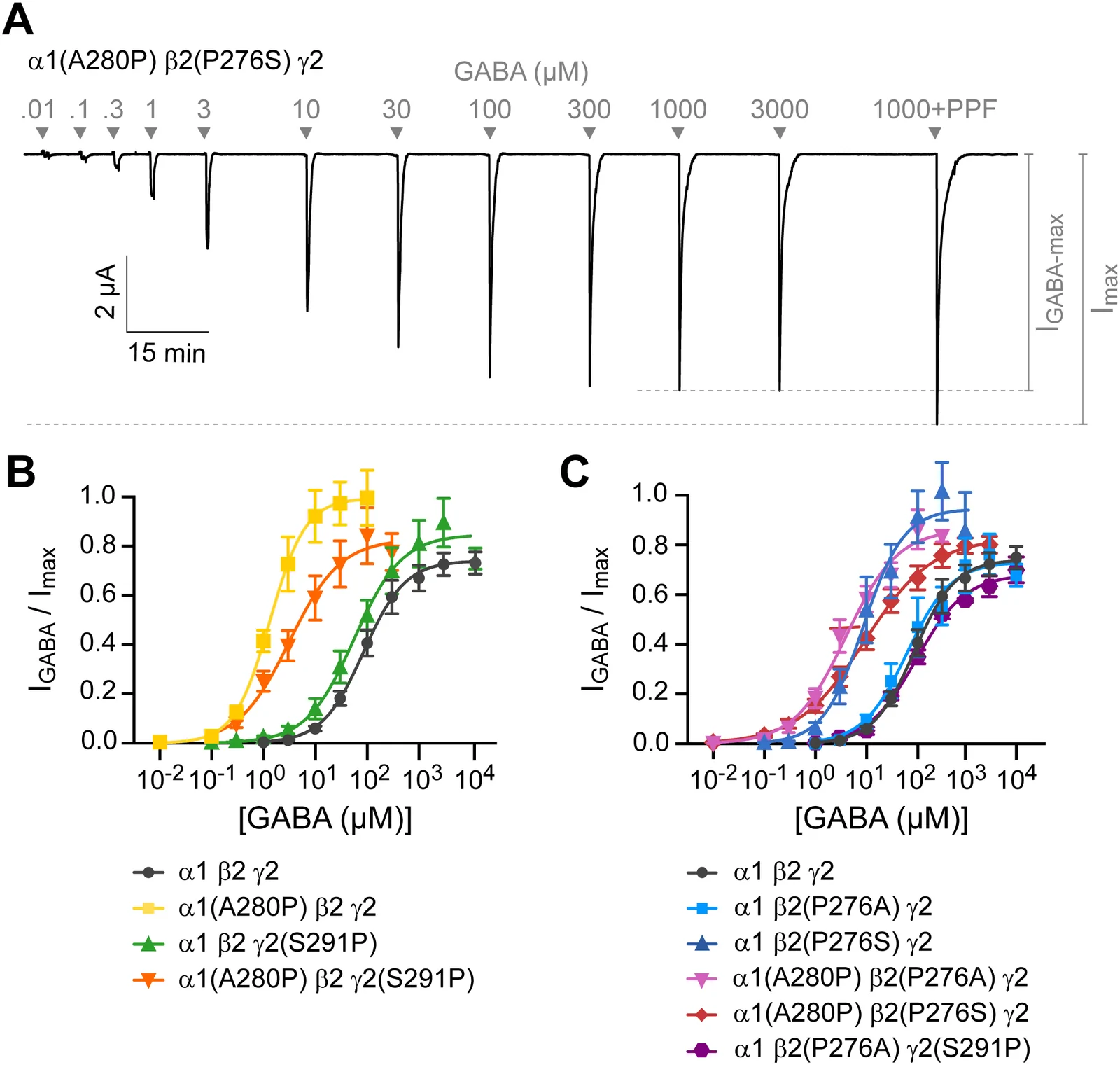
The substitution α1(A280P) promotes channel activation. (A) Representative current tracing of the mutant α1(A280P) β2(P276S) γ2 GABA_A_R construct. Peak current responses to increasing concentrations of GABA (µM) followed by a pulse of saturating GABA in combination with 30 µM propofol (PPF). (B, C) Normalized concentration-response curves for GABA-elicited currents. Curves are the Hill equation fit to the means (see Eq 1). Datapoints are plotted as mean ± SEM. See Table 1 for summary statistics of fit parameters and number of oocytes.

*Xenopus laevis* oocytes were co-injected with cRNA for α1, β2, and γ2 GABA_A_R subunits (either wild type or mutants) in a 1:1:10 ratio [27], and current responses to application of ligands was recorded using two-electrode voltage clamp (see representative current tracings in Fig 3). For each oocyte, we measured current responses to 20–40 s pulses of a series of concentrations of GABA to assess activation sensitivity, and finally the response to the co-application of saturating GABA and propofol to estimate maximal channel activation (Fig 4A). Co-application of saturating GABA and another activator, like propofol, is a typical approach for estimating GABA-evoked maximal open probability [28]. Although this estimation of maximal activation may not reflect conditions where all channels are open with exactly 100% probability, it should be reasonably close. Our logic is that wild-type channels exhibit a maximal open probability of ~0.7–0.8 (70–80%) in saturating GABA [29], which will be increased by addition of propofol. Furthermore, gain-of-function mutations as investigated here will further increase opening. For simplicity, we estimate open probability by assuming it reaches 100% during co-application of saturating GABA and propofol. Importantly, although this approach may slightly overestimate true open probabilities, it will not change our main conclusions regarding enhanced open probability conferred by mutations. Furthermore, using this approach we estimate a peak open probability of 0.74 for wild-type receptors, similar to previous reports [30, 31]. GABA concentration-response curves (CRCs) were normalized to the peak current response elicited by co-application of saturating GABA and propofol and fit with the Hill equation (Eq 1; Fig 4B,C; Table 1).

**Table 1:** Parameters for Hill equation fits to normalized GABA CRCs from Fig 4.

Receptor	${EC}_{50}$ (µM)	H	$I_{GABA}/I_{max}$	n
**α1β2γ2**	86 [75 to 98]	1.08 ± 0.05	0.74 ± 0.01	18
**α1(A280P) β2 γ2**	1.3 [1.3 to 1.4]**	1.26 ± 0.02	1.00 ± 0.01**	4
**α1 β2(P276A) γ2**	62 [40 to 101]	1.00 ± 0.15	0.73 ± 0.03	4
**α1 β2(P276S) γ2**	8.7 [5.4 to 15]**	1.10 ± 0.20	0.95 ± 0.04*	7
**α1 β2 γ2(S291P)**	56 [40 to 81]	0.94 ± 0.11	0.85 ± 0.03	5
**α1(A280P) β2(P276A) γ2**	4.0 [2.4 to 7.6]*	0.86 ± 0.12	0.86 ± 0.04	4
**α1(A280P) β2(P276S) γ2**	9.2 [8.2 to 11]**	0.66 ± 0.02	0.83 ± 0.01**^##^	7
**α1(A280P) β2 γ2(S291P)**	3.2 [2.1 to 5.3]**	0.88 ± 0.11	0.83 ± 0.03^#^	6
**α1 β2(P276A) γ2(S291P)**	95 [68 to 141]	0.91 ± 0.09	0.68 ± 0.02	4

Hill equation is defined in Eq 1. ${EC}_{\text{5}\text{0}}$ (effective concentration 50) is expressed as mean [95% confidence interval], h (Hill coefficient) and $I_{GABA}/I_{max}$ (ratio of saturating GABA-induced current to saturating GABA and propofol current) as mean ± SEM, and n is the number of oocytes. ${log\;EC}_{\text{5}\text{0}}$ and $I_{GABA}/I_{max}$ were analyzed using One-way Brown-Forsythe ANOVA followed by Dunnett's T3 multiple comparisons test.

* p < 0.05, ** p < 0.0001 versus α1β2γ2; ^#^ p < 0.05, ^##^ p < 0.0001 versus α1(A280P)β2γ2.

First, we investigated the effects of introducing a proline at site 2 in the α1 and/or γ2 subunit M2-M3 linker analogous to the naturally occurring proline at the homologous position in the β2 subunit. The substitution α1(A280P) increases GABA sensitivity (i.e., left-shifts the GABA CRC) by ~70-fold (Fig 4B). Furthermore, whereas GABA opens wild type channels with a peak open probability ~0.74, α1(A280P) enhances the efficiency of GABA-activation such that current responses reach a peak open probability of ~1.0 (Fig 4B). In contrast, γ2(S291P) has little effect on channel activation, although there was a small trend towards enhanced activation. The double substitution α1(A280P)β2γ2(S291P) increases GABA sensitivity by ~30-fold, with a similarly modest increase to maximal opening as for γ2(S291P) alone (Fig 4B). Thus, there is some non-additivity in the effects of the double mutant, although α1(A280P) is the more dominant substitution overall. In summary, introduction of a proline at site 2 in the α1 subunit sensitizes the channel for activation by GABA.

### Substituting the site 2 proline in the β2 M2-M3 linker has sidechain-dependent effects on channel activation

We investigated the effects of replacing the site 2 proline in the β2 subunit with the homologous alanine or serine in α1 or γ2 subunits, respectively. The substitution β2(P276S) enhances GABA-sensitivity (i.e., left-shifts the CRC) by ~10-fold and confers an enhanced maximal GABA-elicited peak open probability of ~0.9 (Fig 4C). In contrast, β2(P276A) has no effect on channel activation. Thus, there is a more complex dependence of channel activation on the properties of the sidechain at site 2 in β2 subunits than simply whether it is a proline. We further evaluated double substitutions that effectively move the proline from β2 to α1 or γ2 subunits. Both α1(A280P)β2(P276S)γ2 and α1(A280P)β2(P276A)γ2 receptors show increased GABA-sensitivity and enhanced maximal open probability similar to that of receptors containing either α1(A280P) or β2(P276S) mutations (Fig 4C). Although the underlying mechanism for the differential effects of alanine or serine substitution at β2(P276) are unclear, the observation that introduction of a proline at site 2 in the α1 subunit enhances channel activation is also observed in the tested double substitutions. All combinations that contain the α1 mutant have similarly enhanced gating, irrespective of whether or not a mutation is present in the β2 subunit.

### Introducing a proline at site 2 in the α1 M2-M3 linker prevents channels from remaining closed at rest

**Fig 5:**
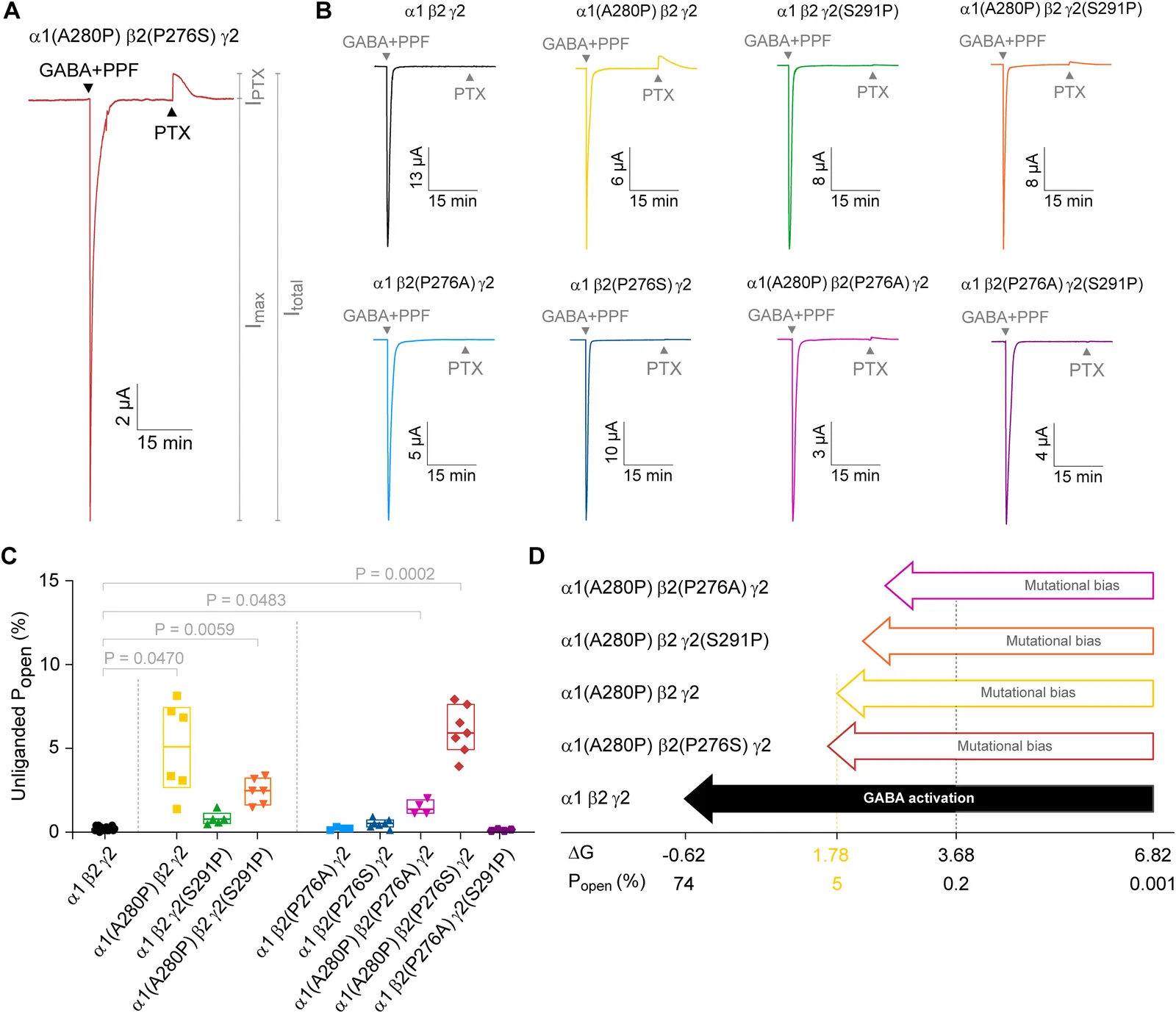
The substitution α1(A280P) confers spontaneously open channels. (A) The total current ($I_{total}$) is estimated as the sum of the peak current response to saturating GABA and 30 µM propofol (PPF; $I_{max}$) and the current blocked by 1 mM picrotoxin (PTX; $I_{PTX}$). (B) Representative tracings of wild-type and mutant α1β2γ2 GABA_A_R constructs showing the peak current response to saturating GABA and 30 µM propofol and PTX-sensitive currents. (C) The unliganded$\;P_{open}$ is estimated as the ratio of PTX-sensitive current to the total current ($I_{PTX}$*/*$I_{total}$). Box plots indicate median and quartiles. *P*-values < 0.05 for Brown-Forsythe ANOVA with posthoc Dunnett’s T3 test are shown. (D) Diagram of estimated closed-open free energy differences (ΔG) for wild type and α1(A280P) containing mutant receptors. Arrows contrast energetic effects of mutations with the energy transduced from GABA binding in wild type receptors. Our experiments place an upper limit on the unliganded $P_{open}$ for wild type receptors of 0.2% (dotted line), but a prior study estimates a much lower unliganded $P_{open}$ of ~0.001% [31]. The black arrow shows the energy transduced from binding of two GABA molecules to opening of the pore in wild type receptors. The other arrows show the estimated energetic changes to the closed-open equilibrium conferred by the indicated mutations in the absence of GABA. See Table 2 for summary statistics and number of oocytes.

To assay for any spontaneous unliganded channel opening in the mutants, we applied the pore blocker picrotoxin (PTX) (Fig 5A,B). The unliganded channel open probability (P_open_) was estimated as the ratio of PTX-sensitive current to the total current from the baseline in PTX to the peak current elicited by the combination of saturating GABA and propofol (Eq 2), as illustrated in Fig 5A and as discussed above. Wild-type channels are essentially closed at rest with an unliganded P_open_ ≤ 0.002 or 0.2% [7, 32]. This is likely an overestimation limited by noise in the recordings, whereas estimates from highly expressing oocytes suggest a much lower unliganded P_open_ of 1 × 10^−5^ (0.001%) [31], which is more consistent with the activation energy estimated from the temperature-dependence of GABA binding [30]. Here, we assume the wild-type unliganded P_open_ to be 1 × 10^−5^. Importantly, our main conclusions do not depend on this approximation and will remain valid even if the unliganded open probability is as high as 0.002. In contrast to wild-type, the substitution α1(A280P) confers obvious PTX-sensitive spontaneous channel activity clearly distinguished from background noise (unliganded P_open_ = 0.05 or 5%) (Fig 5A-C).

To verify that this spontaneous current did not come from a sub-population of either α1(A280P) or β2 homomers [33, 34] that might arise from mutation-induced changes in subunit assembly, we compared oocytes injected with either α1β2γ2, α1(A280P)β2γ2, α1(A280P), or β2 subunits. For each condition, we injected the same amount of RNA per subunit (1 ng each for α and β subunits, 10 ng for γ). For oocytes injected with heteromeric combinations of α1β2γ2 or α1(A280P)β2γ2 subunits we observed robust GABA-evoked responses. In contrast, for oocytes injected with isolated α1(A280P) or β2 subunits, we saw no evidence for any current responses to GABA or propofol, nor any PTX-sensitive spontaneous current (S1 Fig). Thus, we conclude that our observations for site 2 mutants reflect the behavior of heteromeric receptors with α1, β2, and γ2 subunits (or mutants thereof).

All tested combinations of substitutions that included a proline at site 2 in the α1 subunit show noticeable unliganded openings with probabilities of a few percent. The double substitution α1(A280P)β2(P276S)γ2 conferred the most spontaneous channel activity (unliganded P_open_ = 0.06 or 6%) (Fig 5A-C). Although the *P-*values for comparison of spontaneous activity in wild-type versus α1(A280P)β2γ2 and α1(A280P)β2(P276A)γ2 are at the limit of significance, we observe distinct PTX-sensitive currents for the mutants that clearly differ from the background holding current (Fig 5A-C). In contrast, responses to PTX for wild-type receptors are nearly indiscernible from noise. Thus, the effects of α1(A280P) containing mutants on spontaneous channel activity are clearly evident.

Although this unliganded activity of a few percent is rather small in comparison to maximal GABA-evoked opening, it is nonetheless indicative of a relatively sizeable energetic perturbation to the closed-open equilibrium. This is because the energy difference between closed and open states (Eq 3) needs to change appreciably before channel opening can even be reliably detected in two-electrode voltage clamp recordings. For a wild-type channel, we estimate that activation by two GABA molecules results in a change in open probability from 0.001% to 74%, which represents a change in free energy of −7.4 kcal/mol (see Eq 3; Fig 5D; Table 2). This is similar to the −8.9 kcal/mol estimated from the temperature-dependence of GABA binding [30]. Similarly, the energy required to reach an open probability of 5% is −5.0 kcal/mol. Thus, mutants that are spontaneously open with 5% probability in the absence of ligand have shifted the closed-open equilibrium by an amount that is ~ 2/3^rd^ of that induced by GABA binding in wild-type receptors (Fig 5D). Even if we assume that channels start with a higher unliganded P_open_ of 0.2% (an upper limit that likely overestimates the true basal opening), the same logic still suggests that mutants exhibiting only a few percent unliganded activity have shifted the gating equilibrium by ~1/3^rd^ of that induced by GABA binding in wild-type receptors (Fig 5D). As such, even a small observable increase in spontaneous activity represents a significant energetic destabilization of the closed state. Thus, introduction of a proline at site 2 in the α1 subunit prevents the channel from remaining closed at rest.

**Table 2:** Summary of unliganded open probabilities from Fig 5.

Receptor	Unliganded $P_{open}$ (%)	n^†^	ΔG (kcal/mol)	n
**α1β2γ2**	0.21 ± 0.03	18	3.70 ± 0.08	17^‡^
**α1(A280P) β2 γ2**	5.0 ± 1.1*	6	1.86 ± 0.18	6
**α1 β2(P276A) γ2**	0.20 ± 0.04	4	3.72 ± 0.14	4
**α1 β2(P276S) γ2**	0.5 ± 0.1	7	3.26 ± 0.16	7
**α1 β2 γ2(S291P)**	0.8 ± 0.2	4	2.96 ± 0.16	4
**α1(A280P) β2(P276A) γ2**	1.0 ± 0.2*	4	2.52 ± 0.09	4
**α1(A280P) β2(P276S) γ2**	6.0 ± 0.5*	7	1.64 ± 0.06	7
**α1(A280P) β2 γ2(S291P)**	2.0 ± 0.3*	6	2.23 ± 0.09	6
**α1 β2(P276A) γ2(S291P)**	0.15 ± 0.03	4	3.89 ± 0.12	4

Unliganded $P_{open}$ (Eq 2) and ΔG (unliganded closed-open free energy; Eq 3) are expressed as mean ± SEM, and n is the number of oocytes. Unliganded $P_{open}$ was analyzed using One-way Brown-Forsythe ANOVA followed by Dunnett's T3 multiple comparisons test.

* p < 0.05 versus α1β2γ2.

^†^In a few cases, the oocyte count is different from Table 1 because the unliganded $P_{open}$ could not be estimated due to loss of recording stability during the final application of PTX.

‡The oocyte count for ΔG is one less than for $P_{open}$ as one of the $P_{open}$ values was zero, thus precluding a free energy calculation.

## Discussion

We have previously identified M2-M3 linker substitutions in widely expressed heteromeric α1β2γ2 GABA_A_Rs with asymmetric subunit-specific effects. For example, alanine substitution of the central residue in the M2-M3 linker of β2 or α1 subunits either enhances or inhibits channel activation, respectively [6]. Ablation of a main-chain hydrogen bond in the β2 subunit M2-M3 linker also enhances channel activation and prevents the channel from remaining closed at rest, whereas analogous hydrogen bond ablation in the α1 subunit has no effect [7]. Given the location of the β2 subunit M2-M3 linker directly below the GABA binding sites, we hypothesized that M2-M3 linkers at GABA-binding (β2) and non-binding (α1, γ2) interfaces may have distinct functional contributions to channel gating. One difference in the amino acid sequences of M2-M3 linkers is the site 2 position, which is a proline in β subunits and a non-proline residue in all other subunits. Given that prolines in nearby positions within or next to the M2-M3 linker (sites 1 and 3) have been implicated as important determinants of channel gating [24–26], we hypothesized that the naturally occurring proline at site 2 in β subunits may contribute to the distinct functional contribution of the β2 M2-M3 linker in α1β2γ2 receptors.

To test this hypothesis, we performed various substitutions which swapped proline and non-proline residues at site 2 in α1, β2, and γ2 subunits. Although our results do not unambiguously define the functional requirements for the site 2 residue in distinct subunits, they do provide new insight into the molecular basis for channel gating in this region. We show that introduction of a proline at site 2 in α1 subunits 1) sensitizes the channel to activation by GABA, 2) maximizes the efficiency of GABA activation, and 3) prevents the channel from remaining closed at rest.

Injection of RNA encoding either β2 or α1(A280P) subunits alone did not produce GABA_A_-mediated currents (S1 Fig), suggesting that the spontaneous activity we observe with site 2 mutants reflects activity of heteromeric mutant receptors and not a sub-population of constitutively active homomeric receptors. Another possibility is that the α1(A280P)-containing receptors are incapable of co-assembling with γ2 subunits, and the resulting ternary receptors are therefore more sensitive to GABA. However, α1(A280P)-containing receptors show increased GABA-evoked maximal open probabilities and spontaneous activity, whereas binary αβ receptors would exhibit reduced maximal open probability (P_open_ = 0.40–0.49) [35, 36] and no spontaneous activity. Therefore, although we cannot discard the possibility that the mutated α1 subunit is no longer co-assembling with γ2, this alone would not explain our observations.

We conjecture that introduction of a proline-induced kink allows the α1 M2-M3 linker to more easily move radially outward from a naturally more rigid conformation closer to the channel’s central pore axis, thus priming the channel for activation. This idea is consistent with structural observations showing smaller relative motions of α1 versus β2 subunit M2-M3 linkers between closed and activated conformations of wild type α1β2γ2 receptors [37, 38]. The facilitated gating induced by the substitution α1(A280P) may be specific to the proline rigid structure, as substituting the alanine with a serine has been shown to confer a small decrease in GABA-sensitivity [39]. There is also a trend towards enhanced activity upon introduction of proline at site 2 in the γ2 subunit, although the effect is much smaller than in α1. The weaker effect in γ2 could potentially reflect the expectation that the most probable receptor stoichiometry contains two α1 subunits and only one γ2 subunit [40–43]. Substitution of the naturally occurring site 2 proline in the β2 subunit with serine also enhanced GABA-activation, but substitutions with alanine had no effect. Thus, specific sidechain interactions at this position in β2 are important, but proline is not a requirement. In summary, we show that absence of a proline at site 2 in the α1 subunit is important for channels to remain closed at rest. Our observations are consistent with the idea that the backbone geometry of the M2-M3 linker at site 2 in the α1 subunit is an important determinant of the channel’s activation energy landscape.

## Materials and methods

### Mutagenesis and in vitro transcription

DNA for wild-type and mutant GABA_A_R rat α1, β2, and γ2 subunits was subcloned in the pUNIV vector [44]. The mature protein numeration for β2 and γ2 subunits is the same for rat and human, but the numeration for the rat α1 subunit is (human numeration −1) for most of the subunit. Mutations were introduced using QuikChange II (Qiagen) or by GenScript and confirmed by sequencing of the entire subunit. Complementary RNA (cRNA) for each construct was generated (mMessage mMachine T7, Ambion), quantified (Qubit, ThermoFisher Scientific) and quality assessed (TapeStation, Agilent) prior to injection in *Xenopus laevis* oocytes.

### Isolation of *Xenopus laevis* oocytes and injection of cRNA

Defolliculated *Xenopus laevis* oocytes were obtained from EcoCyte Bioscience or harvested from mature female *Xenopus laevis* frogs (Nasco), which were housed in the University of Texas at Austin animal facility. Frog care and surgery followed the Animal Research: Reporting of In Vivo Experiments (ARRIVE) guidelines and the University of Texas at Austin Institutional Animal Care and Use Committee (IACUC-)-approved protocol (approval number AUP-2021–00216). Frogs were anesthetized via immersion in a 3-aminobenzoic acid ethyl ester (Tricaine) solution (0.23% in water), buffered to a neutral pH using sodium bicarbonate, for a total time period of 40 minutes. Frogs were euthanized using an overdose via immersion in a Tricaine solution (0.5% in water), buffered to a neutral pH using sodium bicarbonate, for a total time period of 3 hours, and then decapitated. *Xenopus laevis* oocytes were harvested from frogs under tricaine anesthesia. A piece of ovary was removed from the frog, and placed in isolation media (108 mM NaCl, 2 mM KCl, 1 mM EDTA, 10 mM HEPES, pH = 7.5). Oocytes were manually isolated from the thecal and epithelial layers using forceps and then incubated in a collagenase buffer (0.5 mg/mL collagenase from *Clostridium histolytic,* 83 mM NaCl, 2 mM KCl, 1 mM MgCl_2_, 5 mM HEPES) to remove the follicular layer. Before injections, the defolliculated oocytes were transferred to a sterile incubation solution (88 mM NaCl, 1mM KCl, 2.4 mM NaHCO_3_, 19 mM HEPES, 0.82 mM MgSO_4_, 0.33 mM Ca(NO_3_)_2_, 0.91 mM CaCl_2_, 10,000 units/L penicillin, 50 mg/L gentamicin, 90 mg/L theophylline, and 220 mg/L sodium pyruvate, pH = 7.5). Oocytes were injected with 12 ng of total cRNA for α1, β2, and γ2 subunits (wild-type or mutants) in a 1:1:10 ratio (Nanoject, Drummond Scientific). Oocytes were incubated in the sterile incubation solution at 16 ºC.

### Two-electrode voltage clamp recordings

One to three days post injection, currents from channels expressed in *Xenopus laevis* oocytes were recorded in two-electrode voltage clamp (Oocyte Clamp OC-725C, Warner Instruments), digitized using a PowerLab 4/30 system (ADInstruments) and recorded using LabChart 8 software (ADInstruments). Data was obtained from at least two different batches of oocytes for each experimental group. Oocytes were held at −70 mV and perfused continuously (2 ml/min) with ND96 buffer (96 mM NaCl, 2 mM KCl, 1 mM CaCl_2_, 1 mM MgCl_2_, 5 mM HEPES, pH 7.5) or ND96 buffer containing picrotoxin (PTX), GABA, or GABA & propofol. PTX was diluted from a 0.5 M stock solution in DMSO. GABA was diluted from a 1 M stock solution in double distilled water stored at −80 °C. Propofol was diluted from a 30 mM stock solution in DMSO such that the final solution contained ≤0.1% V/V DMSO. The recording protocol was as follows: a 10 s pulse of 1mM PTX was followed by a series of 20–40 s pulses of increasing concentrations of GABA, a 20 s pulse of saturating GABA in combination with 30 µM propofol (PPF), and a final 10 s pulse of 1mM PTX. Every recording was bookended by applications of PTX to correct for any drift and to identify the zero current baseline. Pulses were sufficiently long to resolve the peak response and inter-pulse intervals were 5–15 minutes to allow washout with buffer and currents to return to baseline. Current traces were baselined by subtracting a spline fit to manually selected baseline regions in each trace. Baselined traces were then normalized by subtracting the zero current level in PTX and then dividing by the peak response in GABA and propofol. Baselining and normalization were done with custom scripts in MATLAB 2024b (MathWorks). Normalized GABA concentration-response curves (CRCs) were fit with the Hill equation:


$$\begin{array}{c} \begin{array}{c} \frac{I}{I_{max}}=\frac{\text{1}}{\text{1}\;+{\;\left(\frac{{EC}_{\text{5}\text{0}}}{\left[GABA\right]}\right)}^{h}} \end{array} \end{array}$$ (1)

where I is the magnitude of the GABA-elicited current, $I_{max}$ is the magnitude of the maximal current response elicited by the co-application of GABA and 30 µM propofol, [GABA] is the GABA concentration, ${EC}_{\text{5}\text{0}}$ is the concentration eliciting a half-maximal response, and h is the Hill slope.

The unliganded channel open probability ($P_{open}$) was estimated as:


$$\begin{array}{c} \begin{array}{c} \;P_{open}\;\sim \;\frac{I_{PTX}}{I_{total}} \end{array} \end{array}$$ (2)

where $I_{PTX}$ is the PTX-sensitive current and $I_{total}$ is the total current from the baseline in PTX to the peak current elicited by the combination of saturating GABA and propofol

The closed-open free energy difference (ΔG) was computed as:


$$\begin{array}{c} \begin{array}{c} \Delta G=-RTln\left(\frac{P_{open}}{\text{1}-P_{open}}\right) \end{array} \end{array}$$ (3)

where R is the gas constant and T is the temperature

### Statistical analysis

Summary data was analyzed using Prism 10 (GraphPad). Symbols and error bars are mean ± SEM, and box plots show median and interquartile intervals. Where applicable, we applied One-way Brown-Forsythe ANOVA followed by Dunnett's T3 multiple comparisons test. We focus on visually evident effects as opposed to relying solely on *P*-value. For example, effect sizes that are multiple time larger than the variation, or clear current block by PTX well beyond small artifacts that can arise from exchanging solutions.

## Supporting information

S1 Fig No GABA_A_-mediated currents after injection of single GABA_A_ receptor subunits. Injection of RNA encoding either β2 or α1(A280P) (1 ng/oocyte) did not elicit spontaneous activity (assessed by application of 1 mM picrotoxin, panel A), nor agonist-evoked currents (maximal GABA concentration, panel B, or maximal GABA concentration + 30 µM propofol, panel C). The maximal GABA concentration was chosen based on the trimeric receptor used for comparison: α1β2γ2 for β2 (3 mM GABA) and α1(A280P)β2γ2 for α1(A280P) (100 µM GABA). In comparison, the trimeric receptors showed robust GABA-evoked currents (panel B) that were enhanced by propofol in the wild-type receptor (panel C). In addition, α1(A280P)β2γ2 receptors exhibited spontaneous activity as assessed by current block with picrotoxin (panel A). All recordings were performed in oocytes from the same batch. Data shown as median ± interquartile range.
(PDF)

S2 Data An excel file containing raw data used to build Fig 4B,C and Fig 5C. (XLSX)
