# Supplementary material for: Introducing a proline in the α1 M2-M3 linker relieves a molecular brake on channel activation in α1β2γ2 GABAA receptors
Source: PLoS One. 2026 Sep 8;21(9):e0357367. doi: 10.1371/journal.pone.0357367 (PMC13552759; doi:10.1371/journal.pone.0357367)
Supplement: Supplementary file 1 [file pone.0357367.s001.pdf]

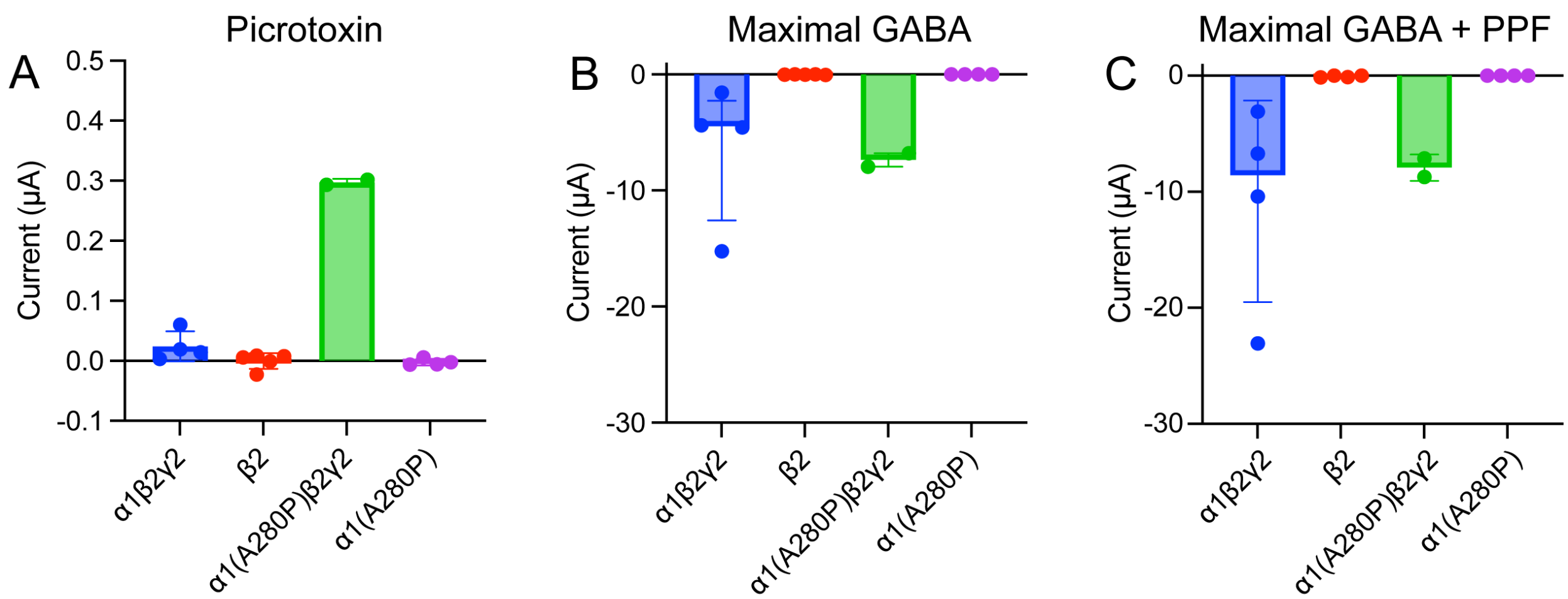

S1 Figure. No $GABA_A$-mediated currents after injection of single $GABA_A$ receptor subunits. Injection of RNA encoding either β2 or α1(A280P) (1 ng/oocyte) did not elicit spontaneous activity (assessed by application of 1 mM picrotoxin, panel A), nor agonist-evoked currents (maximal GABA concentration, panel B, or maximal GABA concentration + 30 µM propofol (PPF), panel C). The maximal GABA concentration was chosen based on the trimeric receptor used for comparison: α1β2γ2 for β2 (3 mM GABA) and α1(A280P)β2γ2 for α1(A280P) (100 µM GABA). In comparison, the trimeric receptors showed robust GABA-evoked currents (panel B) that were enhanced by propofol in the wild-type receptor (panel C). In addition, α1(A280P)β2γ2 receptors exhibited spontaneous activity as assessed by current block with picrotoxin (panel A). All recordings were performed in oocytes from the same batch. Data shown as median ± interquartile range.
