# Supplementary material for: Introducing a proline in the α1 M2-M3 linker relieves a molecular brake on channel activation in α1β2γ2 GABAA receptors
Source: PLoS One. 2026 Sep 8;21(9):e0357367. doi: 10.1371/journal.pone.0357367 (PMC13552759; doi:10.1371/journal.pone.0357367)
Supplement: Supplementary file 2 [file pone.0357367.s004.docx]

We thank the reviewers for their constructive comments, which we believe have helped to improve the manuscript. In this document, we have provided a point-by-point response addressing the reviewers’ comments along with the journal requirements.

**Journal Requirements**

1.Please ensure that your manuscript meets PLOS ONE's style requirements, including those for file naming. The PLOS ONE style templates can be found at

<https://journals.plos.org/plosone/s/file?id=wjVg/PLOSOne_formatting_sample_main_body.pdf> and

[https://journals.plos.org/plosone/s/file?id=ba62/PLOSOne_formatting_sample_title_authors_affiliations.pdf](https://track.editorialmanager.com/CL0/https:%2F%2Fjournals.plos.org%2Fplosone%2Fs%2Ffile%3Fid=ba62%2FPLOSOne_formatting_sample_title_authors_affiliations.pdf/1/010f019d2dcc8280-b85b2aae-5046-439a-9d1c-2f85027fc984-000000/m8slWKQqA909dw_kY3m87MdtTDc_2ezpmp-rPpQqHcY=256)

We have formatted the manuscript according to PLOS ONE’s style requirements.

2. Please note that funding information should not appear in any section or other areas of your manuscript. We will only publish funding information present in the Funding Statement section of the online submission form. Please remove any funding-related text from the manuscript.

We removed all funding-related text from the manuscript.

3. We note that your Data Availability Statement is currently as follows: All relevant data are within the manuscript and in Supporting Information files.

Please confirm at this time whether or not your submission contains all raw data required to replicate the results of your study. Authors must share the “minimal data set” for their submission. PLOS defines the minimal data set to consist of the data required to replicate all study findings reported in the article, as well as related metadata and methods (<https://journals.plos.org/plosone/s/data-availability#loc-minimal-data-set-definition>).

For example, authors should submit the following data:

- The values behind the means, standard deviations and other measures reported;

- The values used to build graphs;

- The points extracted from images for analysis.

We included all individual values used to build the graphs, along with the means, standard deviations, medians and interquartile measures, in an Excel file that is now included in the submission.

4. Please include your full ethics statement in the ‘Methods’ section of your manuscript file. In your statement, please include the full name of the IRB or ethics committee who approved or waived your study, as well as whether or not you obtained informed written or verbal consent. If consent was waived for your study, please include this information in your statement as well.

The full name of the ethics committee who approved our study is included in the manuscript [University of Texas at Austin Institutional Animal Care and Use Committee (IACUC)], along with the specific protocol (approval number AUP-2021-00216) (page 16, lines 299-302).

5. If the reviewer comments include a recommendation to cite specific previously published works, please review and evaluate these publications to determine whether they are relevant and should be cited. There is no requirement to cite these works unless the editor has indicated otherwise.

Although we did not include the studies mentioned by reviewer #2 in minor point #1, we did add a reference that we think applies more closely to our experimental conditions (Keramidas & Harrison, 2010, PMCID: [PMC2806416](https://pmc.ncbi.nlm.nih.gov/articles/PMC2806416/)).

Changes to the references

We have removed the following reference from the manuscript: Hanek, 2009. We believe this reference does not provide any new information compared to other related references. Additionally, we removed the following in-text citation from page 4, line 59: Xiu et al., 2005 (PMID: 16216879). This was incorrectly cited. Changes to the in-text citations are highlighted in the manuscript.

Changes to the tables

When adding new columns to Tables 1-2 we corrected several cases where the number of oocytes were previously incorrect. Note that this has no effect on the reported measures which were correct with one exception. The exception is for Table 2 where the value of the unliganded Popen for the substitution gamma2(S291P) was noticed to include a duplicate value, which we have now corrected.

**Review Comments to the Author**

Reviewer #1: The M23 linker of the LGICs has garnered much attention over the last 2 decades. The proximity of the domain to both the ligand binding and ion channel of the protein have led many to hypothesize that it is a fundamental component of channel gating. This study adds to this body of knowledge.
The manuscript describes well performed assays and is clearly written. It articulates a complex relationship between the M23linker and channel activation.
I have a few questions, some of which can be addressed with additional prose, others that might need more experiments.

1. Adding/removing prolines can alter protein folding and receptor assembly. Consequently, functional heteropentamers lacking the gamma subunit or beta homomers may be expressed in different ratios to those normally observed. Could these effects account for the increased picrotoxin effect, or changes in EC50?

The reviewer raises an interesting point. However, we do not feel that homopentamers are likely, and absence of the gamma2 subunit cannot explain all the observed effects of mutations.

Beta2 subunits do not form homomeric receptors, as they lack the necessary residues in the extracellular domain (Sexton et al., 2021, PMID: 34526505), which are not mutated in our study. Thus, we think it is unlikely that homomers contribute to our observations.

For the receptors to respond to GABA, both the beta2 and alpha1 subunit must be present, so those subunits form part of the receptors in the plasma membrane. However, it is possible that co-assembly with the gamma2 subunit could have been disrupted. Although loss of the gamma2 subunit could explain the left-shift of the EC50 for, e.g., alpha1(A280P)-containing receptors, the other effects (spontaneous opening and increased efficacy) of this mutation cannot be explained by the absence of gamma2 which would predict opposite effects (no spontaneous opening and same or decreased efficacy). A similar argument is made for other mutants with notable effects. As such, we did not test the receptors for the presence of gamma2 subunit, either with a ligand of the benzodiazepine site nor with Zn++.

We have added text to better clarify this (page 14, lines 258-269).

2. The authors make a strong argument for the role the M23 linker plays in gating. The change in EC50 and block by picrotoxin support this argument. However, use of a partial agonist or single channel experiments would provide stronger evidence.

We agree with the reviewer that it would be interesting to explore further the biophysics of these mutated receptors. We considered testing partial agonists, but the vast majority (if not all) of gain-of-function mutations lead to an increased efficacy of the partial agonists, and we did not expect anything different from these receptors. Although single channel measures would provide additional details on gating kinetics, it is unclear that they would clarify the mechanism underlying the increased sensitivity. There is a vast literature that backs the importance of the M2-M3 linker for gating in all the pentameric ligand-gated ion channels, and we think our results illuminate the effect of a proline in the second site of the linker.

3. Proline bestows several unique properties on proteins: constraining protein movement and enabling cis-trans conformations in switches. With so many LGIC structures now available, I was surprised to see that the authors did not attempt to model the new structures the mutant subunits may adopt. Modeling is still an imprecise science but can generate useful hypotheses. Even without the models, I would like the authors to suggest how the mutations alter coupling, structure and function.

We did take advantage of AlphaFold to model the mutated structures. Unfortunately, AlphaFold has not proven very useful for predicting structural changes due to point mutations. In addition, molecular dynamic simulations are challenged to probe time scales needed to observe changes in gating and thus are typically limited to observations of changes in fluctuations. In summary, it is unclear that such structural modeling approaches would provide useful hypotheses without significant effort beyond the scope of this study. Nonetheless, we conjectured that the proline induces a kink that helps the linker move radially outwards from the pore axis (page 15, lines 270-272). Beyond this, we hesitate to speculate.

4. The authors make the interesting statement "Irrespective of P-value, we focus only on relatively large effects." Please quantify large and why p-value can/should be disregarded.

We have reworded this statement to better convey our thinking (page 19, lines 354-357). Beyond that, we would mention that P-values do not necessarily align with effects that are either sizeable or important, a fact that is well understood. However, we agree with the reviewer that our previous statement was very unclear.

Reviewer #2: In the paper entitled "Introducing a proline in the α1 M2-M3 linker relieves a molecular brake on channel activation in α1β2γ2 GABAA receptors” the authors investigated the impact of mutating the so called proline site 2 in the β2 subunits (at the 276 position) and in analogous positions in the α1 and γ2 subunits. They report that α1(A280P) mutation results in enhanced GABA-sensitivity and spontaneous channel activity, whereas the effect of the γ2(S291P) mutation was minor. The impact of the substitution at the site 2 in the β2 subunit depended on the side chain: β2(P276A) had no effect while β2(P276S) increased GABA-sensitivity with a little increase in spontaneous activity. Overall, the authors conclude that the proline site 2 at the β2 subunits and analogous positions at α1 subunit may lead to a significant increase in GABA-sensitivity of the receptor and increased spontaneous activity. The paper provides new and interesting information related to the role of M2-M3 linker, located at the domain interface of GABAAR, known to play a crucial role in the receptor activation process. There are, however, some points that need to be addressed.

Major
1. Although, in general, it is difficult to extract some precise information on the time course of measured currents using 2 electrode VC in oocytes but I am curious if the authors made an attempt to analyze the kinetics of current responses which would indicate some alterations in the receptor gating (saturating [GABA]) or in the agonist binding (low [GABA]). A related technical issue is that the methodology section is lacking description of the perfusion system and its exchange time. The time scale bars in Figs. 3-5 refers to a surprisingly long time: 15 min. Is this a mistake ? In similar experiments in other papers (e.g. [29]) typical scale bars refer to seconds, not minutes.

We have not attempted to analyze the kinetics of the current responses. Our flow rate is 2mL/min, and we cannot increase it, as a faster flow rate would rip the oocyte away from the electrodes in our recording chambers. However, exchange is primarily limited by the large size of the oocyte (~1.2mm diameter), its highly invaginated plasma membrane, and possibly the presence of the vitelline membrane which could contribute to perfusion turbulence. All of this conspire against precise kinetic determinations, which is why we did not attempt any.

Regarding the time scale, we show the entire recording in our figures, encompassing not just the ligand applications (20-40 s) but also the washouts (5-15 min), hence the bars representing such a long time. Pierce et al., 2024 [28] show only submaximal, non-desensitizing GABA responses with very short washouts in between. Unfortunately, they do not mention their flow rate, but their applications and washouts (20-45 s and 2-4 min, respectively), are not that different from our own for those GABA concentrations (40 s and 5 min). Furthermore, we observe very similar GABA concentration-response curves to other studies using oocytes (Sigel et al., 1999; PMID: 10501225, Ramerstorfer et al., 2011; PMID: 21248110, Baumann et al., PMID: 14657175).

2. Two clues may argue that considered mutations may affect the receptor gating: a marked increase the spontaneous activity and also increased IGABA/Imax at saturating [GABA] for some mutants (Fig. 4). This issue requires a more extensive discussion.

We agree with the reviewer, and our conclusion is indeed that the receptor gating is affected by these mutations, as suggested by the increased sensitivity to GABA, increased efficiency of GABA, and increased spontaneous activity (page 14, lines 255-257).

Minor
1. Page 5 line 96 statement that “all channels are open with ~100% probability as described previously [29]”. The assumption that GABAAR reaches open probability of 100 % at saturating [GABA] with propofol should be taken with caution. Bai and Orser (1999) used high resolution recordings and have shown that propofol only slightly (8% but significantly) increased response to saturating [GABA] while the assessments of the maximum open probability in these conditions is in the range 0.5-0.8 (e.g. Jones and Westbrook, 1995; et al., 1999; Perrais and Ropert, 1999; Barberis et al., 2000). These estimations argue against maximum Popen of exactly 1.0. Akk group in paper [29] used oocytes and two electrode VC (as in present paper) which offers limited time resolution for which, before the response reaches the peak, a considerable fraction of receptors desensitize hindering precise estimation of maximum open probability at peak. The methodology of describing dose-responses used by authors is correct and the use of propofol which indeed increases Popen is informative but I would recommend not insisting about maximum Popen of 100 %.

We agree with the reviewer that even with co-application of propofol, the maximal open probability may be a bit less than 100%. However, even with this potential slight overestimation of the open probabilities, none of our conclusions will change. Nonetheless, we have added a paragraph that clarifies this issue and our logic (page 6, lines 96-107).

We thank the reviewer for bringing these references to our attention. However, those studies use hippocampal neurons, and therefore involve a heterogeneous group of GABA-A receptors. We now include a reference where the Popen was determined using single channel recordings in patches from HEK293 cells expressing alpha1beta2gamma2 GABA-A receptors (Popen= 0.81, Keramidas & Harrison, 2010, PMCID: [PMC2806416](https://pmc.ncbi.nlm.nih.gov/articles/PMC2806416/)) (page 6, lines 96-98).

2. In the figure 4 A it should be described for which mutant the traces are presented. Without description, at the first glance, the reader may assume that control recordings (for WT receptors) are shown. Analogous remark for the Fig. 5 A.

We apologize for the lack of clarity. We now mention within the figure the mutants from which the traces were recorded in Figures 4A and 5A.

3. For the sake of completness the value of IGABA/Imax at saturating [GABA] should be provided as for some mutants (e.g. α1(A280P)) this value is clearly increased compared to WT (clearly seen in Fig. 4). For these values the statistical comparison should be made between considered mutants and WT (similarly as for spontaneous activity, Table 2, Fig. 5).

We appreciate the reviewers' suggestion. The value of IGABA/Imax is now provided within Table 1 with the corresponding statistical analysis. The statistical comparison between WT and mutants is now provided in Table 2 and within Fig. 5C. In both cases (IGABA/Imax and spontaneous activity) the analysis used was One-way Brown-Forsythe ANOVA followed by Dunnett's T3 multiple comparisons test.

4. The conclusion presented in the sentence page 8 lines 144-147 that "the observation that introduction of a proline at site 2 in the α1 subunit enhances channel activation is still robustly supported by the tested double substitutions" is not clear. Actually, the impact of the α1 subunit mutation appears not to be influenced by the mutation of the β2 subunit (irrespective of A or S substitution) thus it is not clear what is meant for the "robust support" from double substitutions.

We have now modified the phrase to increase comprehension: "the observation that introduction of a proline at site 2 in the α1 subunit enhances channel activation is also observed in the tested double substitutions. All combinations that contain the α1 mutant have similarly enhanced gating, irrespective of whether or not a mutation is present in the β2 subunit." (page 9, lines 160-163).

5. Page 8, lines 142 and 143. In the description of receptor types γ2 subunit should be added (it looks like α1β2 receptors were studied).

We apologize for the omission. We now include γ2 when we mention the other two subunits (page 9, line 157).

6. There is a semantic issue in the description of the increased spontaneous activity in some of the considered mutants. For instance, in the section title on page 8, lines 148, 149 the information that introduction of proline "prevents channels from remaining closed at rest" is overstated. Data in Fig. 5 actually show a relatively small induction of the spontaneous activity meaning that a minor destabilization of the shut state took place. Probably the authors meant "preventing the channel from remaining permanently closed at rest" but this would be problematic too as WT channels show some (low) spontaneous activity. This statement about “preventing the closed state” is present over and over throughout the manuscript and I would recommend to use a less controversial description of this finding.

We respectfully disagree with the reviewer that a spontaneous activity of ~5% represents a minor destabilization of the shut state. For a wild type channel to transition from fully closed (Po ~0.001%, Campo-Soria et al., 2006; PMID: 16783415) to 5% open requires ~2/3^rd^ of the energy for GABA-activation of wild type channels (i.e., to reach Po ~0.75). Thus, even a small observable spontaneous activity of a few percent represents a destabilization of the closed state by an amount that is a significant fraction of the total chemical energy supplied by GABA binding in wild type channels. We have attempted to clarify this point in the text (pages 11-12, lines 212-225) and have added a panel in Figure 5D to visually depict these energetics. We do agree that even wild type channels open with very low probability in the absence of ligand. However, the mutants we are talking about here open with orders of magnitude higher probability, and thus we feel that our statement that some mutants prevent the channel from remaining closed at rest is warranted. We do not see any semantic difference by adding the word “permanently”. As such, we have left this statement as is.

7. Page 10, lines 173. Although the intention is clear, the statement that PTX "elicits" current is awkward and confusing (although unfortunately sometimes it is used). PTX blocks currents and thereby reveals current mediated by spontaneously active receptors.

We agree with the reviewer and apologize for the oversight. We have changed that phrase to: "current blocked by 1 mM picrotoxin" (page 10, line 192).

8. In Fig. 5 two p values are just at the limit of significance (0.047 and 0.0483) that is a little but disturbing considering also relatively low number of trials.

Even though the p-values are at the limit of significance given the relatively low number of trials (typical in our field), we observed clear and obvious changes in the leaking current when the PTX was applied to those receptors, in stark contrast with what happens in wild type and some of the other mutants, where the effects of PTX were almost indiscernible from noise in the recordings. These clear changes observed in the baseline current in response to the PTX application point to a real effect. To help clarify this, we have added text (page 10, line 182-187) and a new panel Figure 5B (the old Figure 5B is now 5C).

Reviewer #1: **Yes:** Andrew Jenkins

Reviewer #2: No
