# Supplementary material for: Introducing a proline in the α1 M2-M3 linker relieves a molecular brake on channel activation in α1β2γ2 GABAA receptors
Source: PLoS One. 2026 Sep 8;21(9):e0357367. doi: 10.1371/journal.pone.0357367 (PMC13552759; doi:10.1371/journal.pone.0357367)
Supplement: Supplementary file 3 [file pone.0357367.s005.docx]

1. Reviewer #1 original comment: Adding/removing prolines can alter protein folding and receptor assembly. Consequently, functional heteropentamers lacking the gamma subunit or beta homomers may be expressed in different ratios to those normally observed. Could these effects account for the increased picrotoxin effect, or changes in EC50?

Authors’ original response: The reviewer raises an interesting point. However, we do not feel that homopentamers are likely, and absence of the gamma2 subunit cannot explain all the observed effects of mutations.

Beta2 subunits do not form homomeric receptors, as they lack the necessary residues in the extracellular domain (Sexton et al., 2021, PMID: 34526505), which are not mutated in our study. Thus, we think it is unlikely that homomers contribute to our observations.

For the receptors to respond to GABA, both the beta2 and alpha1 subunit must be present, so those subunits form part of the receptors in the plasma membrane. However, it is possible that co-assembly with the gamma2 subunit could have been disrupted. Although loss of the gamma2 subunit could explain the left-shift of the EC50 for, e.g., alpha1(A280P)-containing receptors, the other effects (spontaneous opening and increased efficacy) of this mutation cannot be explained by the absence of gamma2 which would predict opposite effects (no spontaneous opening and same or decreased efficacy). A similar argument is made for other mutants with notable effects. As such, we did not test the receptors for the presence of gamma2 subunit, either with a ligand of the benzodiazepine site nor with Zn++.

We have added text to better clarify this (page 14, lines 258-269).

**Reviewer #1 new response:** I disagree. Sexton et al describes effect of beta2 expression on spontaneous gating in heteromeric receptors. Sexton cites Eaton who in turn cites Cestari who both describe spontaneous activity with beta2 homomers. (PMID: 25238745, 8724679)

**Authors’ new response:** We thank the reviewer for pointing out our mistake, and we acknowledge that there are a couple of reports of spontaneous activity from beta2 homomers. We agree with the reviewer that in this case having a significant population of beta2 homomers with spontaneous activity would potentially impact our interpretation. Thus, we compared oocytes injected with alpha1, beta2, and gamma2 subunits to those injected with only beta2 subunit (new Figure S1). Within the same batch of oocytes, we see robust GABA-evoked currents from oocytes injected with all three subunits, and no GABA-evoked currents from oocytes injected only with beta2 subunit. More importantly, we see no evidence of any PTX-sensitive spontaneous current or propofol-evoked current in oocytes injected only with beta2 subunit. Thus, we conclude that beta2 homomers cannot account for the spontaneous current we observe from mutant receptors, nor are they likely to contribute appreciably to any of our measured responses. To further verify that one of the primary mutations we describe has not somehow given rise to a population of homomeric mutant receptors that are spontaneously active, we also injected oocytes with alpha1(A280P) subunit alone. Analogous to beta2, we saw no evidence for any GABA- or propofol-evoked currents, nor any evidence for any spontaneous PTX-sensitive current in oocytes injected with alpha1(A280P) alone (new Figure S1). Thus, we conclude that our observations reflect heteromeric receptors with alpha1, beta2, and gamma2 subunits (or mutants thereof).

It is worth noting that Eaton et al. (PMID: 8724679) and Cestari et al. (PMID: 25238745) injected either 3- or 50-fold more beta2 subunit RNA than we did, respectively, although it is unclear if this can explain the differences in our observations. Regardless, we do not see any evidence of beta2 homomer spontaneous activity in our hands.

2. Reviewer #1 original comment: The authors make a strong argument for the role the M23 linker plays in gating. The change in EC50 and block by picrotoxin support this argument. However, use of a partial agonist or single channel experiments would provide stronger evidence.

Authors original response: We agree with the reviewer that it would be interesting to explore further the biophysics of these mutated receptors. We considered testing partial agonists, but the vast majority (if not all) of gain-of-function mutations lead to an increased efficacy of the partial agonists, and we did not expect anything different from these receptors. Although single channel measures would provide additional details on gating kinetics, it is unclear that they would clarify the mechanism underlying the increased sensitivity. There is a vast literature that backs the importance of the M2-M3 linker for gating in all the pentameric ligand-gated ion channels, and we think our results illuminate the effect of a proline in the second site of the linker.

**Reviewer #1 new response:** The authors have not addressed my concern.

**Authors new response:** We would ask the reviewer to clarify why the provided evidence which makes a strong argument is insufficient, and exactly what partial agonist or single channel experiments would add and why they are necessary.
