# Supplementary material for: Introducing a proline in the α1 M2-M3 linker relieves a molecular brake on channel activation in α1β2γ2 GABAA receptors
Source: PLoS One. 2026 Sep 8;21(9):e0357367. doi: 10.1371/journal.pone.0357367 (PMC13552759; doi:10.1371/journal.pone.0357367)
Supplement: Peer review history

# Peer review: Decision Letter 0

**DOI:** 10.1371/journal.pone.0357367.r001

**Authors:** Uwe Rudolph

27 Mar 2026

Dear Dr. Goldschen-Ohm,

plosone@plos.org. When you're ready to submit your revision, log on to https://www.editorialmanager.com/pone/ and select the 'Submissions Needing Revision' folder to locate your manuscript file.

- A letter that responds to each point raised by the academic editor and reviewer(s). You should upload this letter as a separate file labeled 'Response to Reviewers'.
- A marked-up copy of your manuscript that highlights changes made to the original version. You should upload this as a separate file labeled 'Revised Manuscript with Track Changes'.
- An unmarked version of your revised paper without tracked changes. You should upload this as a separate file labeled 'Manuscript'.

If you would like to make changes to your financial disclosure, please include your updated statement in your cover letter. Guidelines for resubmitting your figure files are available below the reviewer comments at the end of this letter.

If applicable, we recommend that you deposit your laboratory protocols in protocols.io to enhance the reproducibility of your results. Protocols.io assigns your protocol its own identifier (DOI) so that it can be cited independently in the future. For instructions see: https://journals.plos.org/plosone/s/submission-guidelines#loc-laboratory-protocols. Additionally, PLOS ONE offers an option for publishing peer-reviewed Lab Protocol articles, which describe protocols hosted on protocols.io. Read more information on sharing protocols at https://plos.org/protocols?utm_medium=editorial-email&utm_source=authorletters&utm_campaign=protocols.

As the corresponding author, your ORCID iD is verified in the submission system and will appear in the published article. PLOS supports the use of ORCID, and we encourage all coauthors to register for an ORCID iD and use it as well. Please encourage your coauthors to verify their ORCID iD within the submission system before final acceptance, as unverified ORCID iDs will not appear in the published article. *Only*  the individual author can complete the verification step; PLOS staff *cannot*  verify ORCID iDs on behalf of authors.

We look forward to receiving your revised manuscript.

Kind regards,

Uwe Rudolph

Academic Editor

PLOS One

Journal Requirements:

When submitting your revision, we need you to address these additional requirements.

1.Please ensure that your manuscript meets PLOS ONE's style requirements, including those for file naming. The PLOS ONE style templates can be found at

https://journals.plos.org/plosone/s/file?id=wjVg/PLOSOne_formatting_sample_main_body.pdf and

https://journals.plos.org/plosone/s/file?id=ba62/PLOSOne_formatting_sample_title_authors_affiliations.pdf

2. Please note that funding information should not appear in any section or other areas of your manuscript. We will only publish funding information present in the Funding Statement section of the online submission form. Please remove any funding-related text from the manuscript.

3. We note that your Data Availability Statement is currently as follows: All relevant data are within the manuscript and in Supporting Information files.

Please confirm at this time whether or not your submission contains all raw data required to replicate the results of your study. Authors must share the “minimal data set” for their submission. PLOS defines the minimal data set to consist of the data required to replicate all study findings reported in the article, as well as related metadata and methods (https://journals.plos.org/plosone/s/data-availability#loc-minimal-data-set-definition).

For example, authors should submit the following data:

- The values behind the means, standard deviations and other measures reported;

- The values used to build graphs;

- The points extracted from images for analysis.

Authors do not need to submit their entire data set if only a portion of the data was used in the reported study.

If your submission does not contain these data, please either upload them as Supporting Information files or deposit them to a stable, public repository and provide us with the relevant URLs, DOIs, or accession numbers. For a list of recommended repositories, please see https://journals.plos.org/plosone/s/recommended-repositories.

If there are ethical or legal restrictions on sharing a de-identified data set, please explain them in detail (e.g., data contain potentially sensitive information, data are owned by a third-party organization, etc.) and who has imposed them (e.g., an ethics committee). Please also provide contact information for a data access committee, ethics committee, or other institutional body to which data requests may be sent. If data are owned by a third party, please indicate how others may request data access.

4. Please include your full ethics statement in the ‘Methods’ section of your manuscript file. In your statement, please include the full name of the IRB or ethics committee who approved or waived your study, as well as whether or not you obtained informed written or verbal consent. If consent was waived for your study, please include this information in your statement as well.

5. If the reviewer comments include a recommendation to cite specific previously published works, please review and evaluate these publications to determine whether they are relevant and should be cited. There is no requirement to cite these works unless the editor has indicated otherwise.

[Note: HTML markup is below. Please do not edit.]

Reviewers' comments:

Reviewer's Responses to Questions

**Comments to the Author**

1. Is the manuscript technically sound, and do the data support the conclusions?

Reviewer #1: Yes

Reviewer #2: Yes

**********

2. Has the statistical analysis been performed appropriately and rigorously?

Reviewer #1: Yes

Reviewer #2: Yes

**********

3. Have the authors made all data underlying the findings in their manuscript fully available?

The PLOS Data policy

Reviewer #1: Yes

Reviewer #2: Yes

**********

4. Is the manuscript presented in an intelligible fashion and written in standard English?

Reviewer #1: Yes

Reviewer #2: Yes

**********

Reviewer #1: The M23 linker of the LGICs has garnered much attention over the last 2 decades. The proximity of the domain to both the ligand binding and ion channel of the protein have led many to hypothesize that it is a fundamental component of channel gating. This study adds to this body of knowledge.

The manuscript describes well performed assays and is clearly written. It articulates a complex relationship between the M23linker and channel activation.

I have a few questions, some of which can be addressed with additional prose, others that might need more experiments.

1. Adding/removing prolines can alter protein folding and receptor assembly. Consequently, functional heteropentamers lacking the gamma subunit or beta homomers may be expressed in different ratios to those normally observed. Could these effects account for the increased picrotoxin effect, or changes in EC50?

2. The authors make a strong argument for the role the M23 linker plays in gating. The change in EC50 and block by picrotoxin support this argument. However, use of a partial agonist or single channel experiments would provide stronger evidence.

3. Proline bestows several unique properties on proteins: constraining protein movement and enabling cis-trans conformations in switches. With so many LGIC structures now available, I was surprised to see that the authors did not attempt to model the new structures the mutant subunits may adopt. Modeling is still an imprecise science but can generate useful hypotheses. Even without the models, I would like the authors to suggest how the mutations alter coupling, structure and function.

4. The authors make the interesting statement "Irrespective of P-value, we focus only on relatively large effects." Please quantify large and why p-value can/should be disregarded.

Reviewer #2: In the paper entitled “Introducing a proline in the α1 M2-M3 linker relieves a molecular brake on channel activation in α1β2γ2 GABAA receptors” the authors investigated the impact of mutating the so called proline site 2 in the β2 subunits (at the 276 position) and in analogous positions in the α1 and γ2 subunits. They report that α1(A280P) mutation results in enhanced GABA-sensitivity and spontaneous channel activity, whereas the effect of the γ2(S291P) mutation was minor. The impact of the substitution at the site 2 in the β2 subunit depended on the side chain: β2(P276A) had no effect while β2(P276S) increased GABA-sensitivity with a little increase in spontaneous activity. Overall, the authors conclude that the proline site 2 at the β2 subunits and analogous positions at α1 subunit may lead to a significant increase in GABA-sensitivity of the receptor and increased spontaneous activity. The paper provides new and interesting information related to the role of M2-M3 linker, located at the domain interface of GABAAR, known to play a crucial role in the receptor activation process. There are, however, some points that need to be addressed.

Major

1. Although, in general, it is difficult to extract some precise information on the time course of measured currents using 2 electrode VC in oocytes but I am curious if the authors made an attempt to analyze the kinetics of current responses which would indicate some alterations in the receptor gating (saturating [GABA]) or in the agonist binding (low [GABA]). A related technical issue is that the methodology section is lacking description of the perfusion system and its exchange time. The time scale bars in Figs. 3-5 refers to a surprisingly long time: 15 min. Is this a mistake ? In similar experiments in other papers (e.g. [29]) typical scale bars refer to seconds, not minutes.

2. Two clues may argue that considered mutations may affect the receptor gating: a marked increase the spontaneous activity and also increased IGABA/Imax at saturating [GABA] for some mutants (Fig. 4). This issue requires a more extensive discussion.

Minor

1. Page 5 line 96 statement that “all channels are open with ~100% probability as described previously [29]”. The assumption that GABAAR reaches open probability of 100 % at saturating [GABA] with propofol should be taken with caution. Bai and Orser (1999) used high resolution recordings and have shown that propofol only slightly (8% but significantly) increased response to saturating [GABA] while the assessments of the maximum open probability in these conditions is in the range 0.5-0.8 (e.g. Jones and Westbrook, 1995; et al., 1999; Perrais and Ropert, 1999; Barberis et al., 2000). These estimations argue against maximum Popen of exactly 1.0. Akk group in paper [29] used oocytes and two electrode VC (as in present paper) which offers limited time resolution for which, before the response reaches the peak, a considerable fraction of receptors desensitize hindering precise estimation of maximum open probability at peak. The methodology of describing dose-responses used by authors is correct and the use of propofol which indeed increases Popen is informative but I would recommend not insisting about maximum Popen of 100 %.

2. In the figure 4 A it should be described for which mutant the traces are presented. Without description, at the first glance, the reader may assume that control recordings (for WT receptors) are shown. Analogous remark for the Fig. 5 A.

3. For the sake of completness the value of IGABA/Imax at saturating [GABA] should be provided as for some mutants (e.g. α1(A280P)) this value is clearly increased compared to WT (clearly seen in Fig. 4). For these values the statistical comparison should be made between considered mutants and WT (similarly as for spontaneous activity, Table 2, Fig. 5).

4. The conclusion presented in the sentence page 8 lines 144-147 that "the observation that introduction of a proline at site 2 in the α1 subunit enhances channel activation is still robustly supported by the tested double substitutions" is not clear. Actually, the impact of the α1 subunit mutation appears not to be influenced by the mutation of the β2 subunit (irrespective of A or S substitution) thus it is not clear what is meant for the "robust support" from double substitutions.

5. Page 8, lines 142 and 143. In the description of receptor types γ2 subunit should be added (it looks like α1β2 receptors were studied).

6. There is a semantic issue in the description of the increased spontaneous activity in some of the considered mutants. For instance, in the section title on page 8, lines 148, 149 the information that introduction of proline "prevents channels from remaining closed at rest" is overstated. Data in Fig. 5 actually show a relatively small induction of the spontaneous activity meaning that a minor destabilization of the shut state took place. Probably the authors meant "preventing the channel from remaining permanently closed at rest" but this would be problematic too as WT channels show some (low) spontaneous activity. This statement about “preventing the closed state” is present over and over throughout the manuscript and I would recommend to use a less controversial description of this finding.

7. Page 10, lines 173. Although the intention is clear, the statement that PTX "elicits" current is awkward and confusing (although unfortunately sometimes it is used). PTX blocks currents and thereby reveals current mediated by spontaneously active receptors.

8. In Fig. 5 two p values are just at the limit of significance (0.047 and 0.0483) that is a little but disturbing considering also relatively low number of trials.

**********

what does this mean?). If published, this will include your full peer review and any attached files.

If you choose “no”, your identity will remain anonymous but your review may still be made public.

**Do you want your identity to be public for this peer review?**  For information about this choice, including consent withdrawal, please see our Privacy Policy

Reviewer #1: **Yes:** Andrew Jenkins

Reviewer #2: No

**********

[NOTE: If reviewer comments were submitted as an attachment file, they will be attached to this email and accessible via the submission site. Please log into your account, locate the manuscript record, and check for the action link "View Attachments". If this link does not appear, there are no attachment files.]

To ensure your figures meet our technical requirements, please review our figure guidelines: https://journals.plos.org/plosone/s/figures

You may also use PLOS’s free figure tool, NAAS, to help you prepare publication quality figures: https://journals.plos.org/plosone/s/figures#loc-tools-for-figure-preparation.

NAAS will assess whether your figures meet our technical requirements by comparing each figure against our figure specifications.

# Author comment: Author response to Decision Letter 1

**DOI:** 10.1371/journal.pone.0357367.r002

29 Apr 2026

See file `Response to Reviewers.docx`.

Attachment Submitted filename: Response to Reviewers.docx

# Peer review: Decision Letter 1

**DOI:** 10.1371/journal.pone.0357367.r003

**Authors:** Uwe Rudolph

10 Jun 2026

Dear Dr. Goldschen-Ohm,

Thank you for submitting your manuscript to PLOS ONE. After careful consideration, we feel that it has merit but does not fully meet PLOS ONE’s publication criteria as it currently stands. Therefore, we invite you to submit a revised version of the manuscript that addresses the points raised during the review process.

Please submit your revised manuscript by Jul 25 2026 11:59PM. If you will need more time than this to complete your revisions, please reply to this message or contact the journal office at plosone@plos.org. When you're ready to submit your revision, log on to https://www.editorialmanager.com/pone/ and select the 'Submissions Needing Revision' folder to locate your manuscript file.

- A letter that responds to each point raised by the academic editor and reviewer(s). You should upload this letter as a separate file labeled 'Response to Reviewers'.
- A marked-up copy of your manuscript that highlights changes made to the original version. You should upload this as a separate file labeled 'Revised Manuscript with Track Changes'.
- An unmarked version of your revised paper without tracked changes. You should upload this as a separate file labeled 'Manuscript'.

If you would like to make changes to your financial disclosure, please include your updated statement in your cover letter. Guidelines for resubmitting your figure files are available below the reviewer comments at the end of this letter.

If applicable, we recommend that you deposit your laboratory protocols in protocols.io to enhance the reproducibility of your results. Protocols.io assigns your protocol its own identifier (DOI) so that it can be cited independently in the future. For instructions see: https://journals.plos.org/plosone/s/submission-guidelines#loc-laboratory-protocols. Additionally, PLOS ONE offers an option for publishing peer-reviewed Lab Protocol articles, which describe protocols hosted on protocols.io. Read more information on sharing protocols at https://plos.org/protocols?utm_medium=editorial-email&utm_source=authorletters&utm_campaign=protocols.

As the corresponding author, your ORCID iD is verified in the submission system and will appear in the published article. PLOS supports the use of ORCID, and we encourage all coauthors to register for an ORCID iD and use it as well. Please encourage your coauthors to verify their ORCID iD within the submission system before final acceptance, as unverified ORCID iDs will not appear in the published article. *Only*  the individual author can complete the verification step; PLOS staff *cannot*  verify ORCID iDs on behalf of authors.

We look forward to receiving your revised manuscript.

Kind regards,

Uwe Rudolph

Academic Editor

PLOS One

Journal Requirements:

If the reviewer comments include a recommendation to cite specific previously published works, please review and evaluate these publications to determine whether they are relevant and should be cited. There is no requirement to cite these works unless the editor has indicated otherwise.

Please review your reference list to ensure that it is complete and correct. If you have cited papers that have been retracted, please include the rationale for doing so in the manuscript text, or remove these references and replace them with relevant current references. Any changes to the reference list should be mentioned in the rebuttal letter that accompanies your revised manuscript. If you need to cite a retracted article, indicate the article’s retracted status in the References list and also include a citation and full reference for the retraction notice.

[Note: HTML markup is below. Please do not edit.]

Reviewers' comments:

Reviewer's Responses to Questions

**Comments to the Author**

Reviewer #1: (No Response)

**********

2. Is the manuscript technically sound, and do the data support the conclusions?

Reviewer #1: Yes

**********

3. Has the statistical analysis been performed appropriately and rigorously?

Reviewer #1: I Don't Know

**********

4. Have the authors made all data underlying the findings in their manuscript fully available?

The PLOS Data policy

Reviewer #1: Yes

**********

5. Is the manuscript presented in an intelligible fashion and written in standard English?

Reviewer #1: Yes

**********

Reviewer #1: 1. I disagree. Sexton et al describes effect of beta2 expression on spontaneous gating in heteromeric receptors. Sexton cites Eaton who in turn cites Cestari who both describe spontaneous activity with beta2 homomers. (PMID: 25238745, 8724679)

2. The authors have not addressed my concern.

**********

what does this mean?). If published, this will include your full peer review and any attached files.

If you choose “no”, your identity will remain anonymous but your review may still be made public.

**Do you want your identity to be public for this peer review?**  For information about this choice, including consent withdrawal, please see our Privacy Policy

Reviewer #1: **Yes:** ANDREW JENKINS

**********

[NOTE: If reviewer comments were submitted as an attachment file, they will be attached to this email and accessible via the submission site. Please log into your account, locate the manuscript record, and check for the action link "View Attachments". If this link does not appear, there are no attachment files.]

To ensure your figures meet our technical requirements, please review our figure guidelines: https://journals.plos.org/plosone/s/figures

You may also use PLOS’s free figure tool, NAAS, to help you prepare publication quality figures: https://journals.plos.org/plosone/s/figures#loc-tools-for-figure-preparation.

NAAS will assess whether your figures meet our technical requirements by comparing each figure against our figure specifications.

# Author comment: Author response to Decision Letter 2

**DOI:** 10.1371/journal.pone.0357367.r004

6 Aug 2026

See included Response to Reviewers document.

Attachment Submitted filename: Response_to_Reviewers_auresp_2.docx

# Peer review: Decision Letter 2

**DOI:** 10.1371/journal.pone.0357367.r005

**Authors:** Uwe Rudolph

17 Aug 2026

Introducing a proline in the α1 M2-M3 linker relieves a molecular brake on channel activation in α1β2γ2 GABAA receptors

PONE-D-26-12092R2

Dear Dr. Goldschen-Ohm,

We’re pleased to inform you that your manuscript has been judged scientifically suitable for publication and will be formally accepted for publication once it meets all outstanding technical requirements.

Within one week, you’ll receive an e-mail detailing the required amendments. When these have been addressed, you’ll receive a formal acceptance letter and your manuscript will be scheduled for publication.

An invoice will be generated when your article is formally accepted. Please note, if your institution has a publishing partnership with PLOS and your article meets the relevant criteria, all or part of your publication costs will be covered. Please make sure your user information is up-to-date by logging into Editorial Manager at Editorial Manager® and clicking the ‘Update My Information' link at the top of the page. For questions related to billing, please contact billing support.

If your institution or institutions have a press office, please notify them about your upcoming paper to help maximize its impact. If they’ll be preparing press materials, please inform our press team as soon as possible -- no later than 48 hours after receiving the formal acceptance. Your manuscript will remain under strict press embargo until 2 pm Eastern Time on the date of publication. For more information, please contact onepress@plos.org.

Kind regards,

Uwe Rudolph

Academic Editor

PLOS One

Additional Editor Comments (optional):

Reviewers' comments:

Reviewer's Responses to Questions

**Comments to the Author**

Reviewer #1: (No Response)

**********

2. Is the manuscript technically sound, and do the data support the conclusions?

Reviewer #1: Yes

**********

3. Has the statistical analysis been performed appropriately and rigorously?

Reviewer #1: Yes

**********

4. Have the authors made all data underlying the findings in their manuscript fully available?

The PLOS Data policy

Reviewer #1: Yes

**********

5. Is the manuscript presented in an intelligible fashion and written in standard English?

Reviewer #1: Yes

**********

Reviewer #1: I commend the authors for providing the additional data.

Comparing maximum activation with GABA compared to THIP / Gaboxadol / 4,5,6,7-tetrahydroisoxazolo(5,4-c)pyridin-3-ol or 4-PIOL / 4-piperidinol sulfate / P4S would be a cleaner and more straightforward measure of intrinsic efficacy. Propofol is an excellent choice of course, but at 30uM it has a multitude of actions as a positive modulator and agonist and some of the mutations reported here are quite close to the propofol binding/gating sites. If the authors’ hypotheses are correct, partial agonists like THIP or 4-PIOL would show a bigger reduction in efficacy for gating impaired receptors and improved efficacy in gating enhanced mutants.

**********

what does this mean?). If published, this will include your full peer review and any attached files.

If you choose “no”, your identity will remain anonymous but your review may still be made public.

**Do you want your identity to be public for this peer review?**  For information about this choice, including consent withdrawal, please see our Privacy Policy

Reviewer #1: No

**********

# Editor report: Acceptance letter

**DOI:** 10.1371/journal.pone.0357367.r006

**Authors:** Uwe Rudolph

PONE-D-26-12092R2

PLOS One

Dear Dr. Goldschen-Ohm,

I'm pleased to inform you that your manuscript has been deemed suitable for publication in PLOS One. Congratulations! Your manuscript is now being handed over to our production team.

At this stage, our production department will prepare your paper for publication. This includes ensuring the following:

* All references, tables, and figures are properly cited

* All relevant supporting information is included in the manuscript submission,

* There are no issues that prevent the paper from being properly typeset

You will receive further instructions from the production team, including instructions on how to review your proof when it is ready. Please keep in mind that we are working through a large volume of accepted articles, so please give us a few days to review your paper and let you know the next and final steps.

Lastly, if your institution or institutions have a press office, please let them know about your upcoming paper now to help maximize its impact. If they'll be preparing press materials, please inform our press team within the next 48 hours. Your manuscript will remain under strict press embargo until 2 pm Eastern Time on the date of publication. For more information, please contact onepress@plos.org.

You will receive an invoice from PLOS for your publication fee after your manuscript has reached the completed accept phase. If you receive an email requesting payment before acceptance or for any other service, this may be a phishing scheme. Learn how to identify phishing emails and protect your accounts at https://explore.plos.org/phishing.

If we can help with anything else, please email us at customercare@plos.org.

Thank you for submitting your work to PLOS One and supporting open access.

Kind regards,

PLOS One Editorial Office Staff

on behalf of

Dr. Uwe Rudolph

Academic Editor

PLOS One
